# Effects of Panax Notoginseng Saponins on Esterases Responsible for Aspirin Hydrolysis In Vitro

**DOI:** 10.3390/ijms19103144

**Published:** 2018-10-12

**Authors:** Zongxi Sun, Yali Wu, Song Liu, Shaonan Hu, Bo Zhao, Pengyue Li, Shouying Du

**Affiliations:** 1 School of Chinese Materia Medica, Beijing University of Chinese Medicine, Beijing 100029, China; zongxisun@126.com (Z.S.); wuyali1993@163.com (Y.W.); hushaonan8980@126.com (S.H.); zhaobowua@163.com (B.Z.); 2 College of Medicine, Wuhan University of Science and Technology, Wuhan 430081, China; liusong@wust.edu.cn

**Keywords:** Panax notoginseng saponins, aspirin, HepaRG cells, herb–drug interactions

## Abstract

Herb–drug interactions strongly challenge the clinical combined application of herbs and drugs. Herbal products consist of complex pharmacological-active ingredients and perturb the activity of drug-metabolizing enzymes. Panax notoginseng saponins (PNS)-based drugs are often combined with aspirin in vascular disease treatment in China. PNS was found to exhibit inhibitory effects on aspirin hydrolysis using Caco-2 cell monolayers. In the present study, a total of 22 components of PNS were separated and identified by UPLC-MS/MS. Using highly selective probe substrate analysis, PNS exerted robust inhibitory potency on human carboxylesterase 2 (hCE2), while had a minor influence on hCE1, butyrylcholinesterase (BChE) and paraoxonase (PON). These effects were also verified through molecular docking analysis. PNS showed a concentration-dependent inhibitory effect on hydrolytic activity of aspirin in HepaRG cells. The protein level of hCE2 in HepaRG cells was suppressed after PNS treatment, while the level of BChE or PON1 in the extracellular matrix were elevated after PNS treatment. Insignificant effect was observed on the mRNA expression of the esterases. These findings are important to understand the underlying efficacy and safety of co-administration of PNS and aspirin in clinical practice.

## 1. Introduction

Herbs have been used for primary health care for thousands of years before the advent of modern medicines. It is estimated that approximately 80% of the population in Asian countries currently uses herbs as complementary or alternative medicine [1]. Patients in these countries with complex diseases use herbs more frequently. However, side effects of herb-drug interactions (HDI) might occur. Some research reported that herbs perturbed the activities of the metabolizing enzymes and/or transporters [2,3,4]. In the past decades, concerns about HDI have been raised. Liver is the primary drug metabolism site and entails a myriad of chemical reactions. HepaRG cell line, derived from a hepatocellular carcinoma, could express a large panel of liver-specific enzymes [5,6,7]. Due to the similarity with primary human hepatocytes, HepaRG cell line was found to be a valuable human-relevant in vitro model for testing drug interactions [8].

Panax notoginseng saponins (PNS) are the main biologically active constituents in the roots of Panax notoginseng (family Araliaceae). In China, PNS-based drugs have been developed and widely used to treat cerebral infarction and ischemia [9], coronary heart disease and atherosclerosis [10]. Aspirin (acetylsalicylic acid) is an important member of the therapeutic arsenal for combating the vascular disease. After administration, aspirin is rapidly hydrolyzed in the body with an elimination half-life of approximatly 15 min [11,12]. Aspirin and PNS-based drugs are often taken together to prevent thrombus and clinical benefits have been obtained.

On account of the presence of ester bond, aspirin could be catalyzed by esterases in the intestine, liver, and plasma [13], which mainly include human carboxylesterase 1 (hCE1) and hCE2 in the liver and intestine [14,15], and butyrylcholinesterase (BChE) [16], paraoxonase (PON) [17,18] in plasma. We recently reported that PNS could inhibit aspirin hydrolysis and thereby elevated the level of aspirin through Caco-2 cell monolayers [19]. However, the influence of PNS on the esterases responsible for aspirin hydrolysis has not been fully investigated yet. In the current study, we characterized the effects of PNS on the esterases using highly selective probe substrates and predicted the potential HDI in vivo on the basis of enzyme kinetic parameters of hCE2. The effects toward hCE2 were verified through molecular docking with PNS. Further, we explored the possible HDI using the HepaRG cell line.

## 2. Results

### 2.1. UPLC/LTQ-Orbitrap MS/MS Analysis

**Figure 1:**
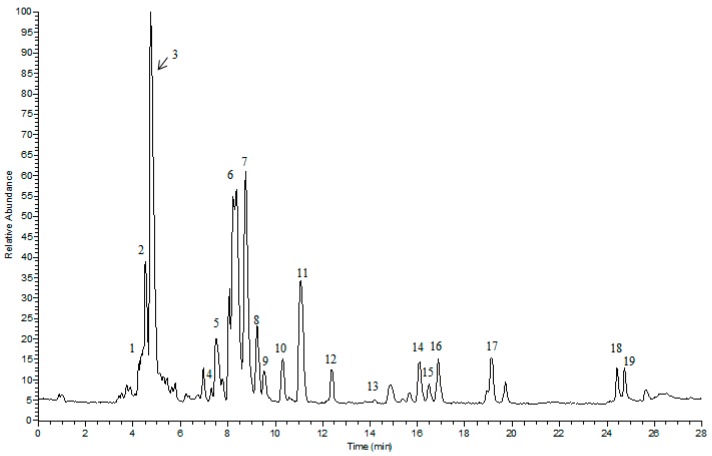
The representative total ion current chromatogram of PNS in negative ESI modes. The corresponding compound names were given in Table 1 (varying from no. 1 to 19).

The main chemical components of PNS were detected with UPLC/LTQ-Orbitrap MS/MS analysis. A total of 22 compounds were identified or tentatively identified (Figure 1). The retention time, *m*/*z* values of adduct ions and MS/MS fragment ions in negative ESI modes, mass error, and formula of all the identified compounds were listed in Table 1.

### 2.2. Enzyme-Specific Probe Substrate Analysis

**Figure 2:**
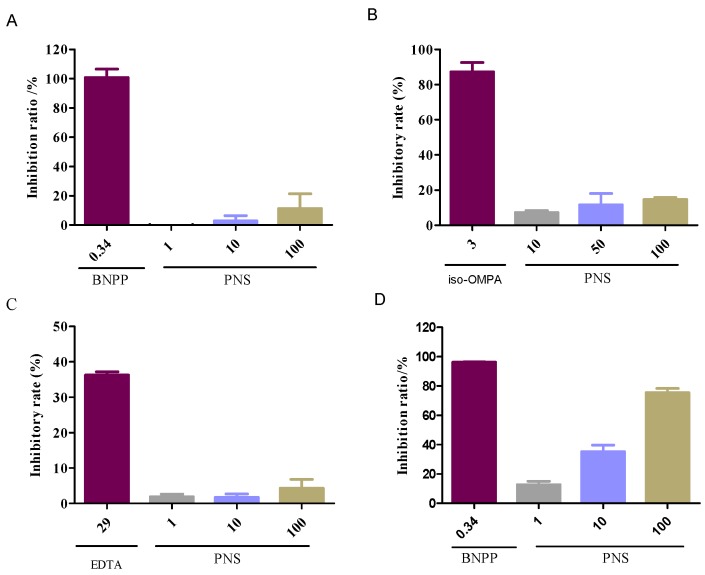
Inhibitory effects of PNS toward the esterases of hCE1, BChE, PON and hCE2 (**A**–**D** in order) with BMBT (2-(2-benzoyloxy-3-methoxyphenyl)benzothiazole), FD (Fluorescein diacetate), BuSCh (S-Butyrylthiocholine chloride) and PA (phenyl acetate) as a highly selective substrate of each esterase. BNPP, iso-OMPA, and EDTA are well-known inhibitors for hCE1 (hCE2), BChE, and PON, respectively. Data were presented as mean ± SD (*n* = 3).

**Figure 3:**
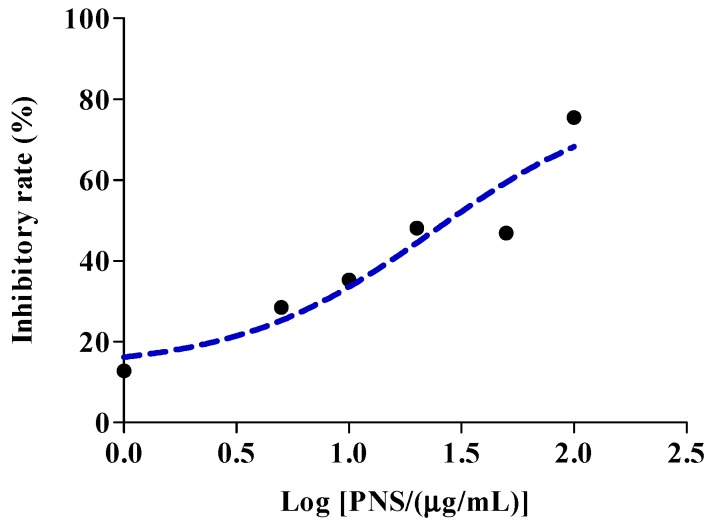
Various concentrations of PNS were used to measure the half inhibition concentration toward hCE2. Each point represents the mean of three independent experiments.

**Figure 4:**
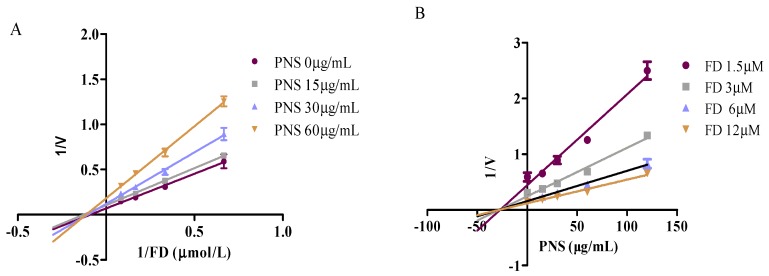
Inhibitory kinetics of PNS toward hCE2 using Lineweaver-Burk plots (**A**) and Dixon plots (**B**). Data were presented as mean ± SD (*n* = 3).

The chemical analysis was conducted to evaluate the influence on the esterases responsible for aspirin hydrolysis. Compared with the positive inhibitors (bis-p-nitrophenyl phosphate, tetraisopropyl pyrophosphoramide and ethylenediaminetetracetic acid, which are abbreviated as BNPP, iso-OMPA and EDTA in order), PNS exhibited weak or negligible effects on hCE1 (Figure 2A), BChE (Figure 2B), and PON (Figure 2C). In contrast, PNS strikingly reduced hCE2 activity in a concentration-dependent manner (Figure 2D). Further, inhibition kinetic analysis was carried out to investigate the mechanism of PNS toward hCE2. The half inhibition concentration (I*C*_50_) of PNS was closed to 23.7 µg/mL (Figure 3). Lineweaver-Burk plots demonstrated that the inhibition type of PNS toward hCE2 was best fit to a non-competitive model (Figure 4A). The inhibition constant *K*_i_ of 27.9 was obtained from Dixon plots (Figure 4B).

A clinical study reported that the integrated peak concentration (*C*_max_) of the top five high-content compounds (notoginsenoside R_1_, ginsenoside Rg_1_, Re, Rb_1_, and Rd with total amounts over 85%) was 33.2 µg/mL after intravenous infusion of Xuesaitong injection (with PNS as an ingredient) at a dosage of 800 mg/d for two weeks [20]. Thus, we figured out that the ratio of the area under the curve (AUC)(+I)/AUC(control) of Xuesaitong Injection was 2.19, indicating that the AUC(+I) caused by PNS toward hCE2 increased by 119% compared with that of without PNS.

### 2.3. Molecular Docking Analysis

**Figure 5:**
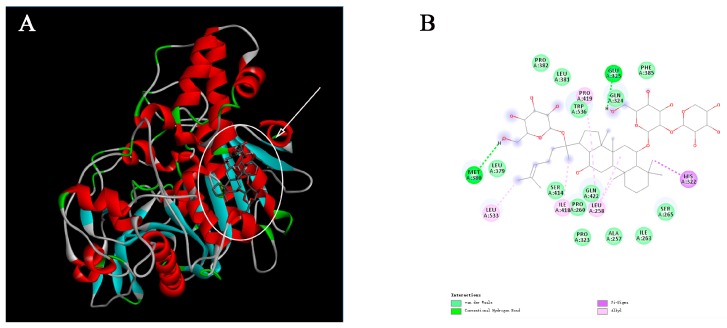

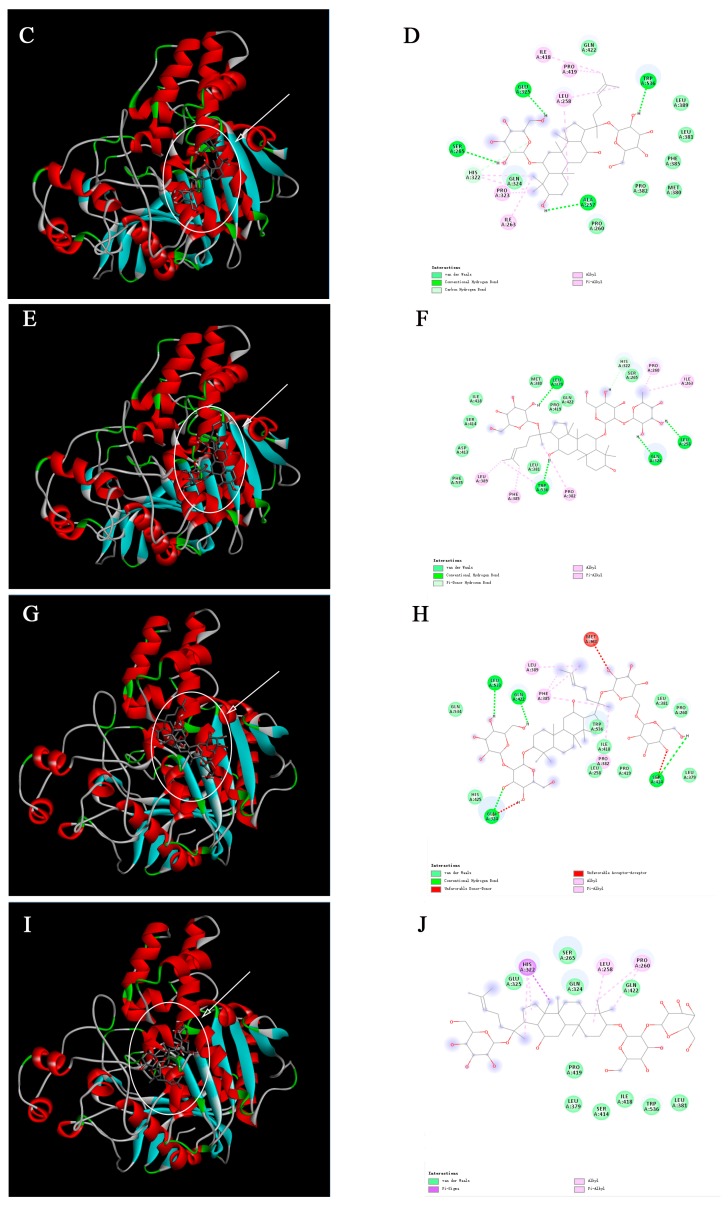
Molecular docking mode and interactions between hCE2 (shown in cartoon representation and colored structure) and notoginsenoside R_1_, ginsenoside Rg_1_, Re, Rb_1_, and Rd (indicated by arrow), respectively. Three-dimensional illustrations show the interactions of hCE2 with notoginsenoside R_1_ (**A**), ginsenoside Rg_1_ (**C**), Re (**E**), Rb_1_ (**G**), and Rd (**I**). Two-dimensional diagrams display interactions of notoginsenoside R_1_ (**B**), ginsenoside Rg_1_ (**D**), Re (**F**), Rb_1_ (**H**), and Rd (**J**) in the active sites of hCE2.

Molecular docking programs use scoring functions to evaluate the binding energy of predicting ligand-receptor complex. The binding scores between PNS and hCE2 were shown in Table 2. The interaction energy between PNS and the metabolic enzyme varied from −8.8 kJ/mol to −5.5 kJ/mol reflecting the stability of the complex. Among 22 compounds in PNS, the five main compounds (notoginsenoside R_1_, ginsenoside Rg_1_, Re, Rb_1_, and Rd) were screened out and found to bind to a few key amino acid residues in the active pocket of hCE2 (Figure 5). Taking notoginsenoside R_1_ as an example, the sugar group formed a conventional hydrogen bond with the residues Glu325 and Met380 to stabilize the space structure of the complex. Moreover, A/B cycle and C-17 side chain were at hydrophobic area and surrounded by residues Leu258, His322, and Leu533.

### 2.4. Cytotoxicology of PNS in HepaRG Cells

**Figure 6:**
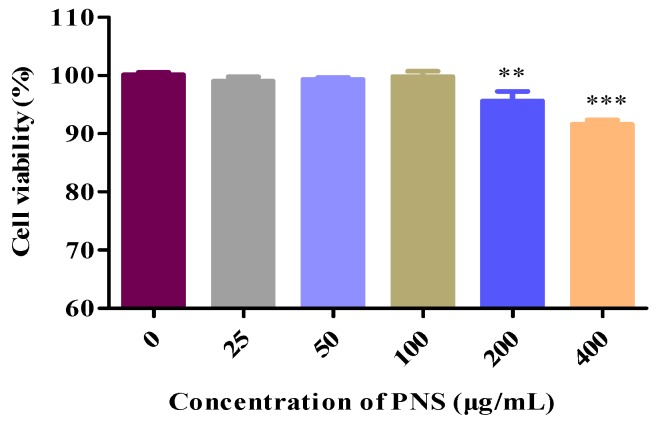
Cytotoxicity tests of PNS on HepaRG cells. Data were presented as mean ± SD (*n* = 5). ** and *** denoted result significantly different from that of the control group (*p* < 0.01 and *p* < 0.001, respectively).

The effects of PNS exposure in HepaRG cells was conducted by an MTT assay to set the concentrations used in the following trials, using the medium-treated cells group as control. As shown in Figure 6, cell viability of PNS-treated cells changed in a concentration-dependent manner and exhibited no cytotoxicity effect under the concentration of 100 µg/mL.

### 2.5. Aspirin Hydrolysis after PNS Treatment

**Figure 7:**
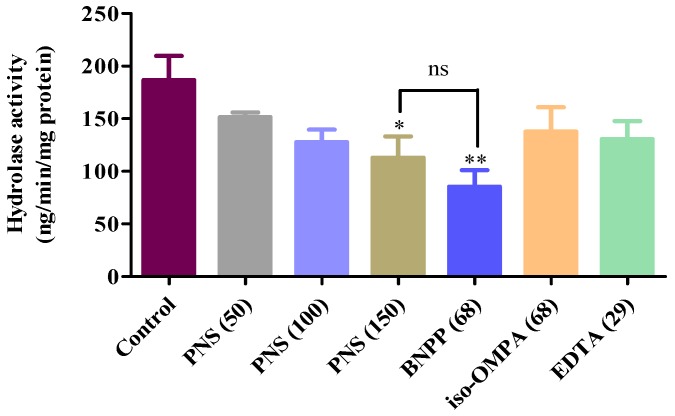
Hydrolysis of aspirin (ASA) in HepaRG cell homogenates treated with PNS. HepaRG cells homogenates were prepared and then diluted with 50 mM HEPES buffer (pH 7.4). Hydrolysis of ASA (1.8 µg/mL) in cell homogenates was conducted in the presence/absence of PNS. No significance (ns) was detected between BNPP (68 µg/mL) and PNS (150 µg/mL) treated group. Data were presented as mean ± SD (*n* = 3). *, and ** denoted results significantly different compared with the control group (*p* < 0.05, *p* < 0.01, respectively).

The property of esterases responsible for aspirin hydrolysis in HepaRG cells was investigated in an inhibition experiment. The results were presented in Figure 7. The hydrolase activity was significantly inhibited by the addition of BNPP, a specific inhibitor of hCE1 or hCE2. However, there was no significant inhibition on aspirin hydrolysis when treated with iso-OMPA or EDTA, hydrolase inhibitors for BChE and PON, respectively.

Meaningfully, PNS significantly decreased the hydrolase activity of aspirin in a concentration-dependent manner. There was no significant difference between BNPP (68 µg/mL) and PNS (150 µg/mL). These data indicated that PNS exhibited an appreciating efficiency in inhibiting aspirin hydrolysis.

### 2.6. ELISA Analysis for Esterases

**Figure 8:**
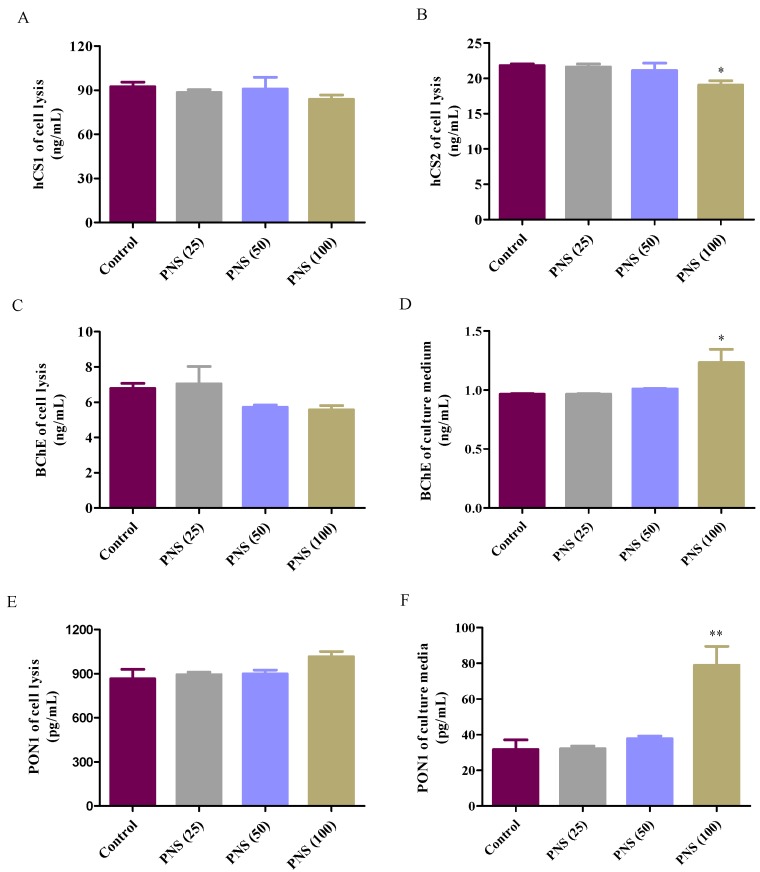
Effects on human carboxylesterase 1 (hCE1) (**A**), hCE2 (**B**), BChE (**C**,**D**), and PON1 (**E**,**F**) protein level after PNS treatment. Cells were incubated with PNS for up to 24 h. After the removal of PNS, cell lysates were prepared for ELISA analysis. Data were presented as mean ± SD (*n* = 3). * and ** denote results significantly different from those of the control group (*p* < 0.05, *p* < 0.01, respectively).

We further explored the role of PNS in the protein expression in HepaRG cells. The results of the expression of hCE1, hCE2, BChE, and PON1 in HepaRG cells after PNS treatment were presented in Figure 8. There was no significant influence on hCE1 expression compared to the control (Figure 8A), while PNS (100 µg/mL) significantly reduced hCE2 protein level (*p* < 0.05) (Figure 8B). Though BChE levels intracelluar was not significantly changed compared to the control, PNS (100 µg/mL) led to the great rise in the extracelluler medium (Figure 8C,D). The same situation was also seen in PON1 (Figure 8E,F).

### 2.7. qRT-PCR Analysis

**Figure 9:**
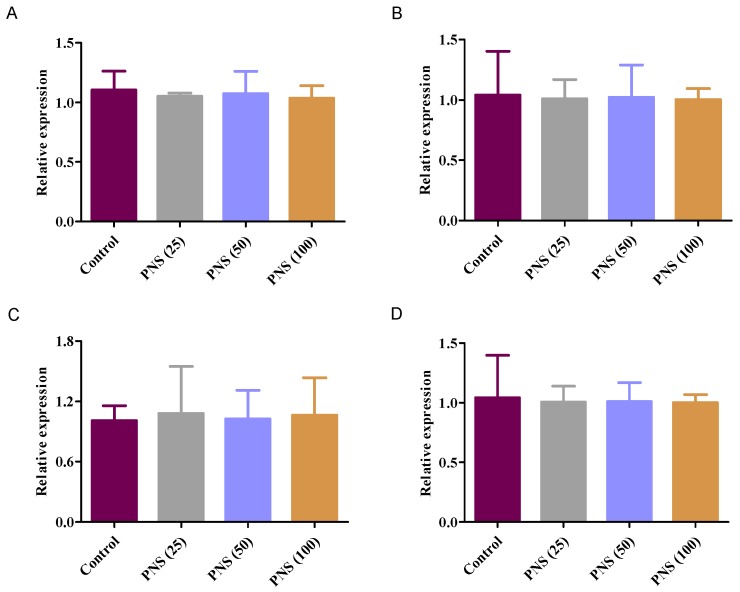
Effects on mRNA expression of human carboxylesterase 1 (hCE1) (**A**), hCE2 (**B**), BChE (**C**), and PON1 (**D**) with PNS treatment. Cells were incubated with PNS for up to 24 h. After the removal of PNS, total mRNA was prepared for qRT-PCR. Data were presented as mean ± SD (*n* = 3).

To elucidate the effect of PNS on the transcription factor contributed to the change of the protein level of esterases, we also explored mRNA expression in HepaRG cells with PNS treatment. Compared with the control, there was no significant difference on the mRNA expression of hCE1, hCE2, BChE, and PON1 (Figure 9).

## 3. Discussion

Multi-drug therapy is now a common therapeutic practice for patients due to their therapeutic benefits, in both developed and developing countries. As a consequence, drug interactions are sometimes unavoidable. Apart from cytochrome P450, esterases is an important class of phase I metabolic enzymes, and plays a vital role in the biotransformation of ester-linked compounds. Drugs with the ability to inhibit the catalytic activity of esterases in the body might improve systemic exposure of the drug which is primarily cleared via the esterases. Except for synthetic compounds, many natural triterpenoids have been reported to exhibit the potent inhibitory effects on esterases in recent years [21,22,23].

Aspirin is one of the oldest antiplatelet agents used for antithrombotic therapy. It is rapidly hydrolyzed to salicylic acid by esterases in the body. Compared with other esterases, it has been reported that aspirin was primarily hydrolyzed by hCE2 [15]. In China, aspirin and PNS-based drugs are often taken together under many conditions and produce enhanced therapeutic effects. In our previous study, the plasma concentration of salicylic acid was highly increased when PNS and aspirin were co-administrated in SD rats, indicating that the HDI in vivo definitely existed in both drugs [24]. In addition, using Caco-2 cell monolayers model, we demonstrated that PNS exhibited appreciative inhibitory ability on the activity of the esterases responsible for aspirin hydrolysis, resulting in the elevated level of aspirin across Caco-2 cell monolayers [19].

Highly-selective probe substrate analysis is extensively used for determination of the inhibitory potential of a test compound. The basic premise of the methodology is that if test compounds cannot inhibit a particular metabolic enzyme, the probe substrate will be metabolized to its product (metabolite) which can then be measured. Conversely, if test compounds inhibit the enzyme, the substrate will not be metabolized or the rate of biotransformation and the formation of product declines [25]. In the study, we used probe substrate analysis to explore the inhibitory effects of PNS toward esterases. The results demonstrated that PNS exhibited strong inhibitory influence toward hCE2, whereas displayed weak inhibition toward hCE1, BChE, and PON with I*C*_50_ higher than 100 µg/mL, along with well-known inhibitors for verification. We provided convincing evidence that the activity of esterases responsible for aspirin hydrolysis could be directly inhibited by PNS treatment with varying potency. Lineweaver–Burk plot analysis showed that the inhibitory pattern of PNS toward hCE2 could be attributed to a non-competitive model. Hence, we speculated that PNS could bind to some specific sites of hCE2 and might alter its original spatial conformation, resulting in the reduced enzymatic activity.

Theoretically, significant enzyme inhibition occurs when the concentration of the inhibitor at the metabolic site is not lower than the *K*_i_ [26]. An apparent *K*_i_ value established in vitro defines the affinity of an inhibitor toward a particular enzyme. The likelihood of an in vivo interactions is projected on the basis of the [I]/*K*_i_ ratio. Generally, the likelihood of interactions in vivo increases along with the ratio increases. The interactions in vivo were considered significant when the ratio of [I]/*K*_i_ > 1 [27]. In the study, we predicted the likelihood of HDI in vivo for Xuesaitong injection based on the inhibitory kinetics data obtained from in vitro studies. High possibility of HDI was found to exist in vivo via moderating hCE2 by PNS. However, since the effect of other metabolic pathways on drug elimination is not taken into account, and the drug clearance is assumed to be mediated only by inhibiting hCE2, the possibility of drug interactions between PNS and aspirin might be overestimated. Applied *C*_max_ as a substitute for [I] might also overestimate the actual HDI effects. Though [I]/*K*_i_ can predict the occurrence of drug interactions, in vivo quantitative prediction on the basis of in vitro investigations should be further confirmed, and studies should be carried out to explore the potential pharmacokinetic interactions in vivo.

Molecular docking is the widely used methods to investigate structure-activity relationships owing to the predictive ability of the conformation of small-molecule ligands within receptor binding sites [28]. By executing quantitative predictions of binding energetics, molecular docking provided rankings of docked compounds on the basis of the binding affinity of complexes [29,30]. The main saponins presented in PNS include notoginsenoside R_1_ and ginsenosides Rg_1_, Re, Rb_1_, and Rd [31]. These ginsenosides contain two to five sugars. The presence of key molecular interactions and the calculated binding free energy were usually used to evaluate the reliability of the predicting enzyme-inhibitor complexes. In the current study, the compounds in PNS demonstrated high binding affinity for hCE2 with the Gibbs free energy. The molecular interactions induced these compounds to anchor in the binding sites of hCE2. Thus, we speculated that some active sites of hCE2 were occupied by these compounds and could alter the original catalytic activity of hCE2. These results provided valuable information on structure-activity relationships between hCE2 and PNS.

Almost all herbal drugs undergo phase I and/or phase II metabolism to yield inactive or active metabolites. Herbal drugs are made of a complex mixture of pharmacological active phytochemicals [32]. This complexity may act as inhibitors or inducers of liver drug enzymes, thus altering the other drug’s concentration in target sites and influencing therapeutic effects [2]. Liver drug enzymes are the common sites of drug interaction in human. HepaRG cell line can express esterases responsible for aspirin hydrolysis. In the inhibition experiment, we observed an interesting phenomenon that the hydrolysis of aspirin was inhibited by PNS. Thus, it is possible that the inhibitory effects of aspirin hydrolysis triggered by PNS occurred as it travels through human liver. The results further support our studies above and are consistent with our earlier finding that PNS is an effective inhibitor of esterases.

We further explored the potential effects of PNS on the major esterases expressed in the human liver. PNS reduced the protein level of hCE2 in HepaRG cells, indicating together regulation of protein expression other than the catalytic activity. PON1 has been reported to be beneficial in preventing atherosclerosis, attributing to its ability to reduce lipid hydroperoxides [33]. Blatter–Garin et al. reported that aspirin application can increase the PON1 activity in plasma [34]. In this study, PNS increased the release of PON1 out of the HepaRG cells. Considering PON1 catalyzing the hydrolysis of aspirin, this may balance the harmful and beneficial actions of PNS. Notably, PNS had insignificant effects on mRNA expression of four tested esterases. Taken together, it was conceived that the effects of PNS might be related to the translation, processing, and stability of the esterases.

The findings from our study clearly indicated that PNS possess high potency in the inhibition of esterases, in particular hCE2. Aspirin hydrolysis inhibited by PNS also had been confirmed on the cellular level. It is likely that PNS could result in HDI in the clinical practice when combined with aspirin. In many case, however, the effects of some drug in vitro and in vivo have no inevitable correlation. A further study is warranted to investigate the HDI of PNS and aspirin in large test animals.

## 4. Materials and Methods

### 4.1. Chemicals

Aspirin was obtained from the National Institute for the Control of Pharmaceutical and Biological Products (Beijing, China). PNS was obtained from Yunnan Sanqi Technology Co., Ltd. (Wenshan, China). PNS contents were determined as: notoginsenoside R_1_, 7.4%; ginsenoside Rg_1_, 26.3%; ginsenoside Re, 3.7%; ginsenoside Rb_1_, 27.7%; ginsenoside Rd, 7.6%. All chemicals were of the highest quality available.

### 4.2. UPLC/LTQ-Orbitrap MS/MS Analysis

UPLC-MS/MS analysis was conducted on an Ultimate 3000 UPLC system (Thermo Fisher Scientific, Waltham, MA, USA) equipped with an LTQ-Orbitrap Elite mass spectrometer (Thermo Fisher Scientific, Waltham, MA, USA). The chromatographic column used was an ACQUICTY UPLC^®^BEH C_18_ column (1.7 µm, 2.1 × 100 mm). A linear gradient elution of 0.1% formic acid aqueous solution (A) and acetonitrile (B) was employed in the analysis (5–30% solvent B for 2 min, 30–50% solvent B for 18 min, and 50–100% solvent B for 8 min). The flow rate and injection volume were maintained at 0.3 mL/min and 3 µL, respectively. The MS conditions were set as follows: sheath gas flow, 40 arb; auxiliary gas flow, 20 arb; spray voltage, 3 kV; source heater temperature, 300 °C; capillary temperature, 350 °C; capillary voltage, 35 V; tube lens voltage, 110 V, and scan range (*m*/*z*), 50 to 2000.

### 4.3. Enzyme-Specific Probe Substrate Analysis

#### 4.3.1. Enzyme Inhibition Experiments

The highly selectively probe substrates were used to evaluate the influence of PNS on esterases responsible for aspirin hydrolysis. The chemical assays for hCE1 and hCE2 were performed according to the methods reported by Wang et al. [35] and Wang et al. [36], respectively. The study on the effects of BChE was conducted using the method by Gulcin et al. [37]. The influence test for PON was conducted by the method of Furlong et al. [38] with some modifications. Briefly, the reaction system contained fresh plasma and freshly prepared paraoxon substrate solution (1 mM) in a total of 200 µL of assay buffer (pH 8.0, 20 mM Tris-HCl, 1 mM CaCl_2_), in the presence/absence of PNS. The assay was conducted at 37 °C and initiated by adding the substrate solution, and the absorbance was continuously monitored at 270 nm for 5 min. A molar extinction coefficient of 1.31 × 10^3^ was used to calculate the activity.

Known inhibitors were run in triplicate as positive controls: BNPP for hCE1 or hCE2, iso-OMPA for BChE, and EDTA for PON. The *K*_i_ value of PNS was further determined with low I*C*_50_ value (<100 µg/mL).

#### 4.3.2. Inhibitory Kinetics Evaluation and In Vitro-In Vivo Extrapolation

The likelihood of HDI in vivo was predicted based on the [I] and *K*_i_ according to the following equation [27]:

AUC(+I)/AUC(control) = 1 + [I]/*K*_i_

in which AUC(+I) and AUC(control) represent the area under the concentration–time curve in the presence/absence of inhibitor, respectively; [I] represents *C*_max_ of inhibitor in the systemic blood.

### 4.4. Molecular Docking Analysis

Molecular docking studies were performed to investigate the binding mode of PNS to hCE2 by using AutodockVina 1.1.2 (http://autodock.scripps.edu/). To date, there is no complete crystal structure of hCE2. Thus, we constructed the model with homology modeling method. The three-dimensional (3D) coordinates of hCE1 (PDB ID, 1MX9; resolution, 2.9 Å) were downloaded from the Protein Data Bank (http://www.rcsb.org/pdb/home/home.do). The 3D structures of PNS were drawn using the ChemBio3D Ultra 12.0 software (Cambridge Soft Corporation, Cambridge, MA, USA). The AutoDock Tools 1.5.6 package (http://mgltools.scripps.edu/documentation/links/autodock) was employed to generate docking input files. The search grid of hCE2 was identified as center_*x*: −1.27, center_*y*: 32.939, and center_*z*: 27.6, with dimensions of size_*x*: 15, size_*y*: 21, and size_*z*: 19. For Vina docking, the default parameters were used unless otherwise stated. The best-scoring pose as judged by the Vina docking score was selected and visually analyzed using the PyMOL 1.5 software (http://www.pymol.org/).

### 4.5. Cell Culture

HepaRG cells were generously provided by Du Yanan (Tsinghua University, Beijing, China). Cells were cultured in growth medium composed of Williams’ E medium supplemented with 12% fetal bovine serum (FBS), 100 U/mL penicillin, 100 µg/L streptomycin, 5 mg/mL insulin, 2 mM glutamax-I, and 50 µM hydrocortisone hemisuccinate under 5% CO_2_/95% humidified air at 37 °C.

### 4.6. Cell Viability Assay

The cell viability was examined by the methyl thiazolyl tetrazolium (MTT) assay. HepaRG cells were seeded into 96-well plates at 5 × 10^3^ cells per well. After 48 h of incubation, the medium was replaced with a fresh medium containing increasing concentrations of PNS. After 24 h, 20 µL MTT (5 mg/mL) solution was added to each well. The MTT solution was discarded and 150 µL DMSO was added after 4 h. The plates were gently shaken for 5 min and the optical density was measured at 490 nm using a microplate reader (Thermo, USA). The cell viability of the untreated control was arbitrarily considered as 100%.

### 4.7. Hydrolysis Experiments

The changes of aspirin hydrolysis in HepaRG cell homogenates after the addition of PNS was investigated. The harvested cells were suspended in ice-cold buffer and then homogenized using an ultrasonic homogenizer under the ice-cold condition. The cell homogenate was diluted with HEPES buffer to the appropriate protein concentrations. PNS was added to the reaction solution and pre-incubated for 5 min. The reaction was then started by adding aspirin and terminated by adding ice-cold methanol. After centrifugation, the supernatants containing acetic acid (final concentration of 2%) were determined using HPLC. The chromatographic separation was achieved using a C_18_ column (4.6 mm × 250 mm, 5 µm). The mobile phase comprised of acetonitrile-water-acetic (29:69:2, *v*/*v*) at a flow rate of 1.0 mL/min. An injection volume of 10 µL was used, and the detection wavelength was set at 276 nm.

### 4.8. ELISA Analysis for Esterases

The expression level of hCE1, hCE2, PON1, and BChE in HepaRG cells treated by PNS was quantitatively measured using ELISA Kit according to the manufacturer’s protocol. Briefly, 2 × 10^5^ cells/well were seeded in a six-well plate for 48 h and then incubated with different concentrations of PNS for another 24 h. The analyzed cells were washed with cold PBS, resuspended in the RIPA lysis buffer, and then centrifuged at 10,000 rpm for 5 min. The resulting supernatants, together with the conditional media collected from HepaRG cells were assayed, and the enzymatic reaction was determined at 450 nm using an automatic microplate reader.

### 4.9. qRT-PCR Analysis

Total RNA was extracted from the cells using the Trizol (CW0581, CWbio, Beijing, China). One microgram of RNA was used to synthesize cDNA using a reverse transcription reagents Kit (CW0744, CWbio, Beijing, China). The qRT-PCR analysis was then carried out using UltraSYBR (with ROX) on Line Gene 9600 Plus qRT-PCR Detection system (Bioer Technology, Hangzhou, China) in the one-step protocol. Reactions were initiated at 95 °C for 10 min, followed by 45 cycles of 95 °C for 15 s and 60 °C for 60 s. Melting curve analysis was performed to confirm the specificity of primers. The relative mRNA expressions of hCE1, hCE2, BChE, and PON1 mRNA were normalized to GAPDH and calculated using the 2^−ΔΔ*C*t^ method. The primers used in the study were listed in Table 3.

### 4.10. Statistical Analysis

Statistical analysis was performed using Prism v5.0 (GraphPad Software Inc., San Diego, CA, USA). Data were presented in the format of the mean ± SD from at least three independent measurements. ANOVA was run to determine significance between groups in the different experiments. A value of *p* < 0.05 was considered to be significant.

## 5. Conclusions

In conclusion, we provided the comprehensive in vitro data that enable us to understand and predict HDI with PNS. PNS could directly affect the activity of esterases in vitro with varying potency with hCE2 being the most susceptible to inhibition. Molecular docking revealed that PNS targeted hCE2 thus leading to its inhibition. We further confirmed the inhibitory potential at the cellular level. Our data also showed that PNS lead to the alteration of the esterase expression level, while exhibited insignificant effects on mRNA expression. The present work provides an insight into the mechanism exploration governing HDI between aspirin and PNS. We hope that the findings will urge us to pay more attention to the underlying safety and efficiency of combined drugs in the clinic.

## Figures and Tables

**Table 1:** All the identified or tentatively identified components of PNS and their UPLC-MS/MS data.

No.	*T*_R_ (min)	Theoretical Mass (*m/z*)	Experimental Mass (*m/z*)	Mass Error (ppm)	MS^2^ Fragment Ions	Formula	Identification
1	4.42	1007.5421	1007.5417	−0.40	799(100), 637(20.49), 475(1.24)	C_48_H_82_O_19_	notoginsenoside R_3_/R_6_
2	4.48	977.5315	977.5316	0.10	799(100), 637(4.13), 475(3.7)	C_47_H_80_O_18_	notoginsenoside R_1_
3	4.73	845.4891	845.4893	0.24	637(34.70), 475(32.28)	C_42_H_72_O_14_	ginsenoside Rg_1_
4	7.30	845.4891	845.4890	−0.12	637(10.01), 475(6.54)	C_42_H_72_O_14_	ginsenoside F_11_
5	8.02	815.4787	815.4785	−0.25	637(52.99), 475(100), 391(1.53)	C_41_H_70_O_13_	notoginsenoside R_2_ or F_3_
6	8.30	1107.5946	1107.5994	4.33	945(100), 783(22.44), 621(16.01), 459(3.33)	C_54_H_92_O_23_	ginsenoside Rb_1_
7	8.62	829.4944	829.4941	−0.36	637(26.69), 475(100), 391(3.34)	C_42_H_72_O_13_	ginsenoside Rg_2_
8	9.24	683.4365	683.4363	−0.29	945(100), 783(35.03)	C_53_H_90_O_22_	ginsenoside Rb_2_ or Rb_3_
9	9.55	1123.5895	1123.5892	0.27	637(100), 475(16.41)	C_36_H_62_O_9_	ginsenoside Rh_1_
10	10.31	683.4365	683.4362	−0.44	637(100), 475(45.43)	C_36_H_62_O_9_	ginsenoside F_1_
11	11.08	991.5472	991.5465	−0.71	783(70.63), 675(32.46), 475(3.7)	C_48_H_82_O_18_	ginsenoside Re
12	12.36	991.5472	991.5465	−0.71	783(100), 621(7.47), 459(5.62), 375(0.86)	C_48_H_82_O_18_	ginsenoside Rd
13	13.76	961.5367	961.5376	0.94	783(0.68), 621(8.38)	C_47_H_80_O_17_	notoginsenoside Fd
14	16.09	665.4259	665.4281	3.31	-	C_36_H_60_O_8_	ginsenoside Rh_4_
15	16.47	829.4947	829.4944	−0.36	715(100), 621(9.06), 459(18.43), 375(9.79)	C_42_H_72_O_13_	ginsenoside Rg_3_
16	16.88	665.4259	665.4261	0.30	-	C_36_H_60_O_8_	ginsenoside Rk_3_
17	19.11	829.4947	829.4944	−0.36	715(100), 621(9.06), 459(18.43), 375(9.79)	C_42_H_72_O_13_	ginsenoside F_2_
18	24.17	667.4416	667.4431	2.25	-	C_36_H_62_O_8_	ginsenoside Rh_2_
19	24.37	811.4838	811.4843	0.62	603(100)	C_42_H_70_O_12_	ginsenoside RK_1_

**Table 2:** Docking scores between the compounds of PNS and hCE2.

Rank	Compound Name	Affinity (kcal/mol)	Rank	Compound Name	Affinity (kcal/mol)
1	ginsenoside Rg_2_	−8.8	12	ginsenoside F_2_	−6.9
2	ginsenoside Rh_1_	−8.7	12	notoginsenoside F_3_	−6.9
3	ginsenoside Rh_4_	−8.6	14	ginsenoside Fe	−6.8
4	ginsenoside F_1_	−8.3	14	ginsenoside Rg_3_	−6.8
5	ginsenoside R_2_	−8.1	16	notoginsenoside R_1_	−6.7
6	notoginsenoside Fd	−7.7	16	ginsenoside Rb_3_	−6.7
7	ginsenoside Re	−7.4	18	ginsenoside Rb_1_	−6.5
8	notoginsenoside R_6_	−7.3	19	ginsenoside Rb_2_	−6.1
9	ginsenoside RK_3_	−7.2	20	ginsenoside F_11_	−6.0
10	ginsenoside RK_1_	−7.1	20	notoginsenoside R_3_	−6.0
10	ginsenoside Rg_1_	−7.1	22	ginsenoside Rd	−5.5

**Table 3:** Primer sequences for qRT-PCR.

Gene	Forward Sequences 5′–3′	Reverse Sequences 5′–3′
hCE1	GGGCACTGTGATTGATGGGA	CCTTCGGAGAGTGGATAGCTC
hCE2	TCAGAAGTTTCTTTGGGGGCA	GCAGGTATTGCTCCTCCTGG
BChE	GCTGGCCTGTCTTCAAAAGC	TCCTGCTTTCCACTCCCATTC
PON1	AAGTTCGAGTGGTGGCAGAA	TGGCATCCAACCCAAAGGTC
GAPDH	CTCCTCCACCTTTGACGCTG	TCCTCTTGTGCTCTTGCTGG

## References

[1] Choi Y.H., Chin Y., Kim Y.G. (2011). Herb-drug interactions: Focus on metabolic enzymes and transporters. Arch. Pharm. Res..

[2] Brantley S.J., Argikar A.A., Lin Y.S., Nagar S., Paine M.F. (2013). Herb-drug interactions: Challenges and opportunities for improved predictions. Drug Metab. Dispos..

[3] Li C., Wang Q., Ren T., Zhang Y., Lam C.W.K., Chow M.S.S., Zuo Z. (2016). Non-linear pharmacokinetics of piperine and its herb-drug interactions with docetaxel in Sprague-Dawley rats. J. Pharm. Biomed. Anal..

[4] Murray J., Picking D., Lamm A., McKenzie J., Hartley S., Watson C., Williams L., Lowe H., Delgoda R. (2016). Significant inhibitory impact of dibenzyl trisulfide and extracts of Petiveria alliacea on the activities of major drug-metabolizing enzymes in vitro: An assessment of the potential for medicinal plant-drug interactions. Fitoterapia.

[5] Aninat C., Piton A., Glaise D., Le Charpentier T., Langouet S., Morel F., Guguen-Guillouzo C., Guillouzo A. (2006). Expression of cytochromes P450, conjugating enzymes and nuclear receptors in human hepatoma HepaRG cells. Drug Metab. Dispos..

[6] Kanebratt K.P., Andersson T.B. (2008). Evaluation of HepaRG cells as an in vitro model for human drug metabolism studies. Drug Metab. Dispos..

[7] Hart S.N., Ye L., Nakamoto K., Subileau E., Steen D., Zhong X. (2010). A comparison of whole genome gene expression profiles of HepaRG cells and HepG2 cells to primary human hepatocytes and human liver tissues. Drug Metab. Dispos..

[8] Andersson T.B., Kanebratt K.P., Kenna J.G. (2012). The HepaRG cell line: A unique in vitro tool for understanding drug metabolism and toxicology in human. Expert. Opin. Drug. Metab. Toxicol..

[9] Liu L., Zhu L., Zou Y., Liu W., Zhang X., Wei X., Hu B., Chen J. (2014). Panax notoginseng saponins promotes stroke recovery by influencing expression of Nogo-A, NgR and p75NGF, in vitro and in vivo. Biol. Pharm. Bull..

[10] Wan J., Lee S.M., Wang J.D., Wang N., He C.W., Wang Y.T., Kang J.X. (2009). Panax notoginseng reduces atherosclerotic lesions in apoE-deficient mice and inhibits TNF-α-Induced endothelial adhesion molecule expression and monocyte adhesion. J. Agric. Food Chem..

[11] Levy G. (1978). Clinical pharmacokinetics of aspirin. Pediatrics.

[12] Levy G. (1981). Comparative pharmacokinetics of aspirin and acetaminophen. Arch. Intern. Med..

[13] Williams F.M., Mutch E.M., Nicholson E., Wynne H., Wright P., Lambert D., Rawlins M.D. (1989). Human liver and plasma aspirin esterase. J. Pharm. Pharmacol..

[14] Inoue M., Morikawa M., Tsuboi M., Ito Y., Sugiura M. (1980). Comparative study of human intestinal and hepatic esterases as related to enzymatic properties and hydrolizing activity forester-type drugs. Jpn. J. Pharmacol..

[15] Tang M., Mukundan M., Yang J., Charpentier N., LeCluyse E.L., Black C., Yang D., Shi D., Yan B. (2006). Antiplatelet agents aspirin and clopidogrel are hydrolyzed by distinct carboxylesterases, and clopidogrel is transesterificated in the presence of ethyl alcohol. J. Pharmacol. Exp. Ther..

[16] Zhou G., Marathe G.K., Hartiala J., Hazen S.L., Allayee H., Tang W.H., McIntyre T.M. (2013). Aspirin hydrolysis in plasma is a variable function of butyrylcholinesterase and PAF acetylhydrolase 1b2. J. Biol. Chem..

[17] Santanam N., Parthasarathy S. (2007). Aspirin is a substrate for paraoxonase-like activity: Implications in atherosclerosis. Atherosclerosis.

[18] Jaichander P., Selvarajan K., Garelnabi M., Parthasarathy S. (2008). Induction of paraoxonase 1 and apolipoprotein A-I gene expression by aspirin. J. Lipid. Res..

[19] Sun Z.X., Wu Y.L., Yang B., Zhu B.C., Hu S.N., Lu Y., Zhao B., Du S.Y. (2018). Inhibitory influence of Panax notoginseng saponins on aspirin hydrolysis in human intestinal Caco-2 cells. Molecules.

[20] Li X., Wang G., Xiong Y., Sun J., Hao H., Zou L., Zheng Y., Yan B., Xia C., Liu G. (2007). Population pharmacokinetic and pharmacodynamic evaluation of Xuesaitong Injection, a typical multiple constituent tradition Chinese medicine in patients with cerebral ischemia. Chin. J. Clin. Pharmacol. Ther..

[21] Zou L.W., Dou T.Y., Wang P., Lei W., Weng Z.M., Hou J., Wang D.D., Fan Y.M., Zhang W.D., Ge G.B. (2017). Structure-activity relationships of pentacyclic triterpenoids as potent and selective inhibitors against human carboxylesterase 1. Front. Pharmacol..

[22] Mai Z.P., Zhou K., Ge G.B., Wang C., Huo X.K., Dong P.P., Deng S., Zhang B.J., Zhang H.L., Huang S.S. (2015). Protostane triterpenoids from the rhizome of *Alisma orientale* exhibit inhibitory effects on human carboxylesterase 2. J. Nat. Prod..

[23] Jamila N., Khairuddean M., Yeong K.K., Osman H., Murugaiyah V. (2015). Cholinesterase inhibitory triterpenoids from the bark of *Garcinia hombroniana*. J. Enzyme Inhib. Med. Chem..

[24] Tian Z.H., Pang H.H., Du S.Y., Lu Y., Zhang L., Wu H.C., Guo S., Wang M., Zhang Q. (2017). Effect of Panax notoginseng saponins on the pharmacokinetics of aspirin in rats. J. Chromatogr. B.

[25] Wienkers L.C., Heath T.G. (2005). Predicting in vivo drug interactions from in vitro drug design data. Nat. Rev. Drug Discov..

[26] Li T., Li N., Guo Q., Ji H., Zhao D., Xie S., Li X., Qiu Z., Han D., Chen X. (2011). Inhibitory effects of wogonin on catalytic activity of cytochrome P450 enzyme in human liver microsomes. Eur. J. Drug Metab. Pharmacokinet..

[27] Blanchard N., Richert L., Coassolo P., Lavé T. (2004). Qualitative and quantitative assessment of drug-drug interaction potential in man, based on Ki, IC_50_ and inhibitor concentration. Curr. Drug Metab..

[28] Ferreira L.G., Santos R.N.D., Oliva G., Andricopulo A.D. (2015). Molecular docking and structure-based drug design strategies. Molecules.

[29] López-Vallejo F., Caulfield T., Martínez-Mayorga K., Giulianotti M.A., Houghten R.A., Nefzi A., Nefzi A., Houghten R.A., Medina-Franco J.L. (2011). Integrating virtual screening and combinatorial chemistry for accelerated drug discovery. Comb. Chem. High Scr..

[30] Huang S.Y., Zou X. (2010). Advances and challenges in protein-ligand docking. Int. J. Mol. Sci..

[31] Liu H.F., Yang J., Du F.J., Gao X.M., Ma X.T., Huang Y.H., Xu F., Niu W., Wang F., Mao Y. (2009). Absorption and disposition of ginsenosides after oral administration of Panax notoginseng extract to rats. Drug Metab. Dispos..

[32] Hermann R., von Richter O. (2012). Clinical evidence of herbal drugs as perpetrators of pharmacokinetic drug interactions. Planta Med..

[33] Durrington P.N., Mackness B., Mackness M.I. (2001). Paraoxonase and atherosclerosis. Arterioscl. Throm. Vas..

[34] Blatter-Garin M.C., Kalix B., De Pree S., James R.W. (2003). Aspirin use is associated with higher serum concentrations of the anti-oxidant enzyme, paraoxonase-1. Diabetologia.

[35] Wang D.D., Jin Q., Hou J., Feng L., Li N., Li S.Y., Zhou Q., Zou L.W., Ge G.B., Wang J.G. (2016). Highly sensitive and selective detection of human carboxylesterase 1 activity by liquid chromatography with fluorescence detection. J. Chromatogr. B.

[36] Wang J., Williams E.T., Bourgea J., Wong Y.N., Patten C.J. (2011). Characterization of recombinant human carboxylesterases: fluorescein diacetate as a probe substrate for human carboxylesterase 2. Drug Metab. Dispos..

[37] Gulcin I., Scozzafava A., Supuran C.T., Koksal Z., Turkan F., Cetinkaya S., Bingöl Z., Huyut Z., Alwasel S.H. (2016). Rosmarinic acid inhibits some metabolic enzymes including glutathione S-transferase, lactoperoxidase, acetylcholinesterase, butyrylcholinesterase and carbonic anhydrase isoenzymes. J. Enzyme Inhib. Med. Chem..

[38] Furlong C.E., Richter R.J., Seidel S.L., Motulsky A.G. (1988). Role of genetic polymorphism of human plasma paraoxonase/arylesterase in hydrolysis of the insecticide metabolites chlorpyrifos oxon and paraoxon. Am. J. Hum. Genet..

